# Equity Arguments in News Reporting on School Nutrition Policy

**DOI:** 10.1089/heq.2017.0061

**Published:** 2018-06-01

**Authors:** Liana B. Winett, Lori Dorfman, Larissa Yoshino, Laura Nixon

**Affiliations:** ^1^ Oregon Health & Science University - Portland State University School of Public Health, Portland, Oregon.; ^2^ Berkeley Media Studies Group, Berkeley, California.

**Keywords:** child health, framing, health disparities, health equity, health policy, school nutrition

## Abstract

**Purpose:** In two related studies, we examined how equity-based arguments featured in news debate over federal school nutrition policy.

**Methods:** We conducted content analyses of national and local print and broadcast news (September 1, 2014–December 31, 2015), examining arguments rooted in appeals about equity and/or disparities.

**Results:** Equity and/or disparities appeals appeared in 24% television, 14% national print, and 5% local print stories. Socioeconomic inequities were mentioned most; racial/ethnic inequities appeared minimally.

**Conclusions:** Neither equity nor disparity featured prominently in this news debate over policy created to address children's nutritional inequities. When included, arguments focused on overcoming inequities' effects rather than addressing root causes.

## Purpose

Federal policies governing the provision and nutritional quality of school meals have been, since their inception in the 1930s, designed to ensure that all children, irrespective of financial means, have access to the nutrition needed to sustain them during the school day.^1^ This continuous focus on providing nutritional support for all children in need gives these policies an equity aim.

In 2010, these policies were reconceptualized as the Healthy Hunger-Free Kids Act (HHFKA), including both expanded food access standards and more stringent guidelines to increase the nutritional content of foods sold in the nation's nearly 100,000 public schools. The new policy maintained the equity-promoting orientation of prior laws by providing assurances for low-income students' access to free and reduced price school breakfasts and lunches, while also increasing student accessibility to these programs and simplifying administrative processes in districts with high proportions of eligible children.^2^ Speaking to the intent of the law, the then United States Department of Agriculture Secretary, Tom Vilsak, referred to HHFKA as part of the “critical nutrition and hunger safety net for millions of children.”^3^

The HHFKA was controversial almost from its inception. The policy was altered by rollbacks and waivers throughout its first 5 years of implementation and continuing through its scheduled September 2015 reauthorization. Debate over the policy, still awaiting reauthorization >2 years later, has played out in the nation's print and broadcast news. In two related studies, we asked whether the equity-promoting intent of the policy was reflected in news coverage of HHFKA during the highly controversial period of pending reauthorization.^4,5^ This is important, because a policy debate can easily be circumscribed to particular details of implementation without informing the broader public of how the policy reduces disparities or addresses equity. In particular, we asked how HHFKA's core function of reducing children's nutritional disparities was expressed, and whether it, or equity, appeared as the central theme that the policy's history would suggest. Given the policy's focus on ameliorating the effects of socioeconomic inequities on children's nutritional status, and the concentrations of student eligibility and enrollment among children of color,^6^ we wondered whether these variables would appear in news coverage.

## Methods

To detect patterns in news arguments,^7–10^ we conducted two coordinated content analyses of HHFKA coverage from September 1, 2014 to December 31, 2015, a period preceding the anticipated reauthorization and the interval after its initial reauthorization delay. In study 1, we analyzed traditional news coverage and opinion pieces in 4 newspapers with national circulation; 16 television (TV) stations including national network, national cable news programming, and local network affiliates in 2 large and diverse media markets (San Francisco and Philadelphia); and public radio. In study 2, we analyzed traditional news coverage and opinion pieces in 63 local and regional newspapers, spanning 11 states selected to represent different regions, demographic makeups, and state-level policy action in response to HHFKA. In total, the two studies captured reporting on HHFKA reauthorization across a breadth of local and national print and electronic news media, nationwide.

We searched print and public radio stories online using the Lexis/Nexis and ProQuest databases, and TV news using the Internet Archives platform. Both studies used search strings including terms capturing aspects of HHFKA (e.g., “school lunch,” “school breakfast,” “school meals,” “smart snacks,” “competitive foods,” “free and reduced price,” “school nutrition,” “healthy hunger free kids act”), along with indicators of its related outcomes (e.g., “nutrition,” “nutritious,” “obesity,” or “overweight”). Both studies used the same coding parameters, iteratively developed by both research teams to identify arguments addressing sources of, and solutions for, equity and disparity.^11^

Specifically, we looked for *disparity arguments* defined as problem statements characterizing differences in children's nutritional access, and indicated by language expressing quantitative or experiential differences in food availability, nutritional status, or related disease or risk experience. We also coded *equity arguments*, defined as those linking the problem of disparity to specific solutions to resolve or minimize the effects of unequal access or opportunity and indicated by language proposing, appealing to, or describing strategies to remedy these differences.^†^ When we identified disparity or equity arguments, we further analyzed the data for the focus of that argument (e.g., socioeconomic, geographic, and racial/ethnic) as presented in the story.

Three trained coders, each, in study 1 (national newspapers, TV, and public radio) and study 2 (state/local newspapers) analyzed the samples using a FileMaker Pro database custom designed for the studies. All coders were professional public health communication analysts or graduate students. We assessed intercoder reliability within teams using Krippendorf's α, achieving acceptable scores of 0.84 for TV, 0.77 for national newspapers, and 0.81 for state/local newspapers.^12^

## Results

The majority of stories across all media types were traditional news reports (40% print and 84% TV in study 1, 100% in study 2). The remaining stories were opinion formats (i.e., Letter to the Editor, Op-Ed, TV interview, or news roundtable). Because of the small number of radio stories and similarities in reporting style and depth to national newspapers, we collapsed the national print news sample and National Public Radio reporting into a single category, presented here as “national print.” Additional descriptors of the news samples in these two studies are presented in Tables 1 and 2.

**Table 1.** **Sample Descriptors in Study 1 (September 1, 2014–December 31, 2015)**

Type of source	News source	Count of stories
National print news	*The New York Times* *Wall Street Journal* *Washington Post* *USA Today*	54
Network & cable news	ABC, NBC, CBS, and PBS ABC, NBC, CBS local affiliates in San Francisco & Philadelphia CNN, MSNBC, and FOX^*^	80
National Public Radio	NPR	4
Total		134

^*^ We also searched for stories broadcast on CNBC but found none that met the criteria for a substantive focus on federal school foods policy.

**Table 2.** **Sample Descriptors in Study 2 (September 1, 2014–December 31, 2015)**

State print news source	Count of stories
California	14
Illinois	10
Iowa	5
Kansas	9
Massachusetts	13
Michigan	12
Mississippi	4
Oklahoma	6
Texas	12
Washington	6
West Virginia	2
Total	93

**FIG. 1.**
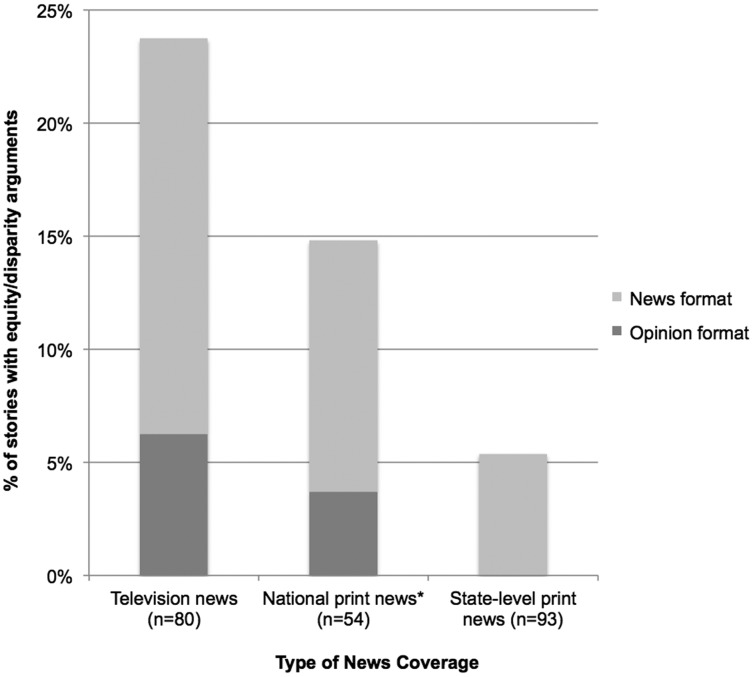
Presence of equity or disparity arguments in news coverage of Healthy Hunger-Free Kids Act reauthorization, September 2014–December 2015 (*n*=227). Television news includes stories broadcast on network stations ABC, CBS, NBC, PBS, local affiliates of ABC, CBS, and NBC in San Francisco and Philadelphia, and cable stations CNN, MSNBC, and FOX. National print news includes stories printed in *The New York Times*, *Wall Street Journal*, *Washington Post*, and *USA Today*, as well as National Public Radio broadcasts. State-level print news includes stories printed in 63 local and regional newspapers, spanning 11 states selected to represent different regions, demographic makeups, and state-level policy actions. News format: stories produced or written in typical news report format. Opinion format: stories produced or written with substantial opinion content including Letter to the Editor, Op-Ed, television interview, or news roundtable discussion formats. *Because of the small number of public radio stories and similarities in reporting style and depth to national newspapers, we collapsed the national print news sample and National Public Radio reporting into a single category.

We found HHFKA discussed in terms of equity or disparity arguments in 24% of TV, 14% of national print, and 5% of local print news stories. Of these stories, roughly a quarter of the TV and national print were in opinion formats (study 1), but none of the local print sample was opinion pieces (study 2; Fig. 1). This relative infrequency of opinion-based pieces in print news stories discussing equity or disparity across both studies is notable because letters and op-eds, in particular, offer advocates and other public health professionals clear opportunities to enter and help shape the debate.

The predominant type of inequity described in the news across both studies was socioeconomic. Language used to describe children affected included “low-income,” “poor children,” and “living in poverty,” although less frequently there was also mention of geographic disparity (e.g., “in some areas,” “high-poverty districts,” and “urban school districts”). Neither study found specific mention of racial or ethnic inequities, although there was a tendency in the TV stories to mention or describe socioeconomic disparity with visuals of children of color.

Solutions presented in stories across both studies mentioned equity-focused efforts to help children overcome the results of disparities (e.g., poor nutrition and inadequate food access), but not strategies to address the systemic or cultural roots of it.

## Discussion

News coverage of the HFFKA rarely included arguments rooted in equity or disparity despite the fact that the HFFKA and its predecessor policies were developed to address nutritional inequities in children. When some form of equity or disparity was mentioned, it was generally socioeconomic and to a lesser extent geographic; arguments were not related to race or ethnicity with the exception of TV's tendency to provide visual references to children of color against a voice describing socioeconomic status.

One reason that race and ethnicity were not expressly discussed in news coverage of the HHFKA reauthorization debate may be that policy advocates serving as news sources in these stories were first emphasizing the universal appeal^13^ of the policy over focusing on specific population groups. However, that advocates and others spoke of the effects of the policy and not the deeper roots of nutritional and health inequities may also reflect reporters' tendency to ask about the immediate circumstances surrounding particular policy events^14^ rather than delving into historical context, such as connections to the entrenched and systemic causes of inequities.

There are some limitations to this analysis. First, although the two studies were designed together, their conduct coordinated, and intercoder reliability established within teams, we did not test coders across teams. Second, these studies represent a specific period of time in HHFKA's trajectory, but may not reflect the entirety of debate surrounding school food policy. Because both of these studies were conducted at the critical reauthorization stage, however, we believe advocates and others would have marshaled the range of what they believed to be their most important and persuasive arguments in support of the policy.

## Conclusions

Each study illuminated the fact that the equity argument was largely missing from debate around HFFKA. By not mentioning the equity origins of the policy—and further, not mentioning the breadth of sources of inequity—news coverage may be obscuring the importance of those factors in this and the larger societal narrative about the social determinants of health.

To help foster broad dialogue about this issue, advocates working on child nutrition and other public health policies to create equitable health environments, and the reporters covering those policy debates, will need to be explicit about the policies' implications for reducing nutritional disparities and furthering health equity.

## Notes

^†^ All search strings and coding criteria are available upon request.
